# A global phylogenetic framework for *Diospyros* L. (Ebenaceae, Ericales): enhanced taxon and sample coverage through NGS, Sanger sequencing and herbariomics

**DOI:** 10.1093/aob/mcag084

**Published:** 2026-04-08

**Authors:** Nattanon Meeprom, Alexander Linan, Sutee Duangjai, Carmen Puglisi, Kálmán Könyves, Timothy Utteridge, Alastair Culham

**Affiliations:** Department of Botany, Faculty of Science, Kasetsart University, Chatuchak, Bangkok 10900, Thailand; Herbarium, School of Biological Sciences, University of Reading, Reading RG6 6EX, UK; Royal Botanic Garden Edinburgh, Edinburgh EH3 5LR, UK; Royal Botanic Gardens, Kew, Richmond TW9 4AE, UK; Missouri Botanical Garden, St. Louis, MO 63110, USA; Department of Forest Biology, Faculty of Forestry, Kasetsart University, Chatuchak, Bangkok 10900, Thailand; Missouri Botanical Garden, St. Louis, MO 63110, USA; Royal Horticultural Society Garden Wisley, Woking GU23 6QB, UK; Royal Botanic Gardens, Kew, Richmond TW9 4AE, UK; Singapore Botanic Gardens, Singapore 259569, Singapore; Herbarium, School of Biological Sciences, University of Reading, Reading RG6 6EX, UK

**Keywords:** *Diospyros*, Ebenaceae, ebony, Ericales, *Euclea*, herbariomics, herbarium specimens, next-generation sequencing, nucleotide diversity, phylogenetics, plastome, *Royena*, Sanger sequencing, *ycf1*

## Abstract

**Background and Aims:**

*Diospyros* (∼700 species) is a pan-tropical genus with centres of diversity in poorly studied areas. Fragmented regional classifications have led to confusion in the understanding of this economically important tropical genus. With <20 % of species and only six plastid loci previously sampled (*atpB*, *ndhF*, *rbcL*, *trnK–matK*, *trnL–trnF* and *trnS–trnG*), existing phylogenies have been poorly resolved and supported. Next-generation sequencing (NGS) provides the possibility of broad sampling of herbarium specimens. In this study, the three main aims are to explore methods for integrating legacy Sanger and new NGS sequence data to make more comprehensive datasets; to build the most complete and well-supported phylogenies of *Diospyros* to date, and to identify the most informative genes for Sanger-based phylogeny building.

**Methods:**

Approximately 200 *Diospyros* and outgroup species, totalling 324 samples, were *de novo* sequenced with NGS technology. One hundred and eleven newly collected samples resulted in 62 whole plastomes and 49 additional partial sequences while 213 herbarium samples generated 48 whole plastomes and 165 additional partial sequences of the six loci. DNA sequences from a further 207 samples from 138 taxa were added from public databases. Single-locus, multi-locus and whole plastome phylogenies were reconstructed using a maximum likelihood approximation method.

**Key Results:**

The six-locus tree elucidates the genus-wide relationships and the whole plastome phylogeny resolves multiple previously ambiguous relationships with robust support. There remain only two unresolved clades, possibly results of rapid radiation and/or hybridization. Whole-plastome analysis shows *ycf1* to be the best-resolving gene when combined with the six legacy loci.

**Conclusions:**

We provide a well-resolved phylogeny for *Diospyros* offering a major advance in understanding the evolution of the genus. The integration of whole-plastome NGS data and legacy chloroplast sequence data enabled us to generate the most comprehensive view of evolutionary relationships for the genus to date (∼250 *Diospyros* taxa).

## INTRODUCTION

Advances in phylogenetic knowledge of life depends on wide taxonomic and geographic sampling along with sufficient depth of data to robustly resolve relationships. In this paper, we sample from key Asian sampling darkspots (Ondo *et al.*, 2024) using next-generation sequencing (NGS) to guarantee good data depth and taxonomic coverage to resolve relationships in the ebony genus, *Diospyros*.

**Fig. 1.**
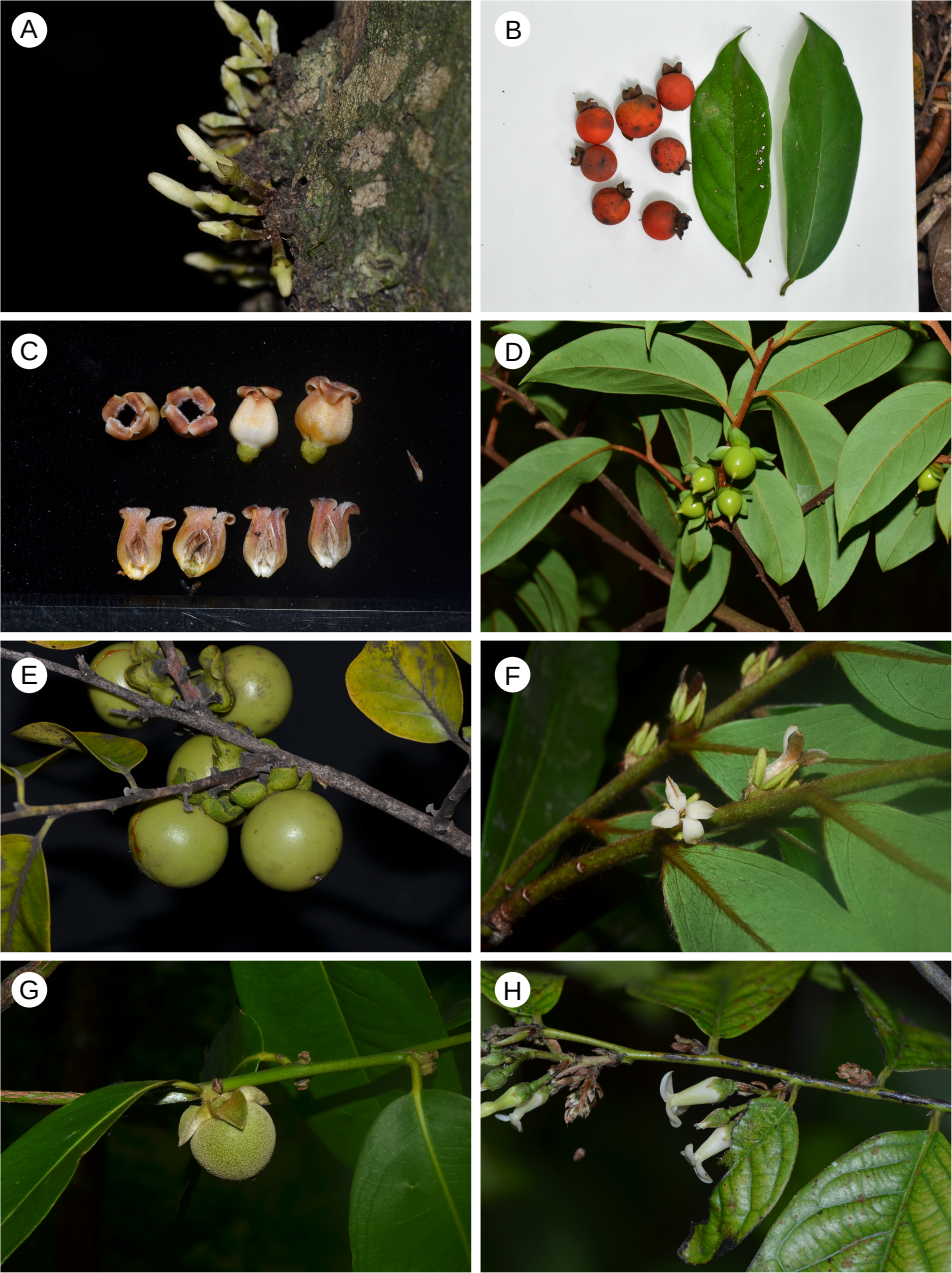
Some Southeast Asian *Diospyros* species with sample ID presented in this paper. (A) *D. brandisiana* (NM204); (B) *D. defectrix* (NM217); (C) *D. hayatae* (NM149B); (D) *D. kerrii* (NM172); (E) *D. mollis* (NM100); (F) *D. rufogemmata* (NM120); (G) *D. siamensis* (NM157); (H) *D. tahanensis* (NM214). Photographs by N. Meeprom.

The genus *Diospyros* (Ebenaceae) (Fig. 1), with over 700 species (Frodin, 2004; Bánki *et al.*, 2025), is considered the 30th largest flowering plant genus and includes some economically important species (Singh, 2005). Persimmon (*D. kaki*) is a globally important economic fruit mostly produced in China, Korea and Japan. Other important fruit-producing species include North American persimmon (*D. virginiana*), black sapote (*D. nigra*), and date plum (*D. lotus*) (Rauf *et al.*, 2017). Many *Diospyros* species produce valuable and durable ebony wood, such as the Indonesian Macassar ebony (*D. celebica*), the Central African forest ebony (*D. crassiflora*), the Ceylon ebony (*D. ebenum*), the Australian Queensland ebony (*D. humilis*), the Vietnamese mun ebony (*D. mun*) and the Pacific blackwood (*D. samoensis*) (Swain, 1928; Anisi *et al.*, 2021; Deblauwe, 2021; Hamalton *et al.*, 2021). However, some of these species are declining due to overexploitation (Kiding Allo, 2020; Deblauwe *et al.*, 2025). Additionally, as of October 2025, over half of the members of the genus have been listed as threatened in the IUCN Red List.

The taxonomy of *Diospyros* is notoriously complex when using morphology alone (Meeprom *et al.*, 2024; Mestre Serra *et al.*, 2024), and genetic data have been proven useful for guiding classification and identification of the genus (Duangjai *et al.*, 2006; Turner *et al.*, 2013*a*; Duangjai *et al.*, 2018). However, phylogenetic studies of this genus highlight the multiple challenges of historic poor sampling, legacy Sanger sequence datasets and patchily resolved phylogeny with some evidence of rapid radiations. There is limited knowledge of the phylogenetic relationships between taxa, with relatively few studies that include broad sampling of taxa (Duangjai *et al.*, 2009; Turner *et al.*, 2013*a*, 2016; Linan *et al.*, 2019; Huang *et al.*, 2023). Most of the phylogenetic studies that aimed to resolve deeper taxonomic relationships within the family Ebenaceae and the genus *Diospyros* utilized Sanger DNA sequencing to generate four to six plastid markers (*atpB*, *ndhF*, *rbcL*, *trnK* intron (including *matK*), *trnL–trnF* (including *trnL* intron and *trnL–trnF* spacer) and *trnS–trnG* spacer) (Duangjai *et al.*, 2006, 2009; Turner *et al.*, 2013*a*; Linan *et al.*, 2019). Whole plastomes have only recently been utilized with a small set of species in New Caledonia (Turner *et al.*, 2016) and China (Huang *et al.*, 2023), whereas in some of the most species-diverse regions, such as Southeast Asia and Africa, relationships within *Diospyros* have not yet been extensively investigated with this approach. To date, the most comprehensive genus-wide phylogeny was compiled by Turner *et al.* (2013*a*) with a set of four plastid loci (*atpB*, *rbcL*, *trnK* intron and *trnS–trnG*) and two additional nuclear markers (*ncpGS* and *PHYA*). Although their study focused on New Caledonian *Diospyros* (∼30 spp.), many species from Africa (∼35 spp.), the Americas (∼20 spp.) and Asia (∼60 spp.) were also included in their phylogenetic analyses. Eleven to 12 clades of *Diospyros* were defined in these previous studies, well supported either by morphology or geography. However, these four to six loci did not provide enough information to resolve a recently diverged New Caledonian species within Clade III (Fig. 1 in Duangjai *et al.*, 2009; Fig. 4 in Turner *et al.*, 2013*a*), and even when whole-plastome data were included the relationships within this clade remained unclear (Turner *et al.*, 2016). Finally, the placement of some clades (Clades VIII, IX and X) was unstable, with the backbone of Clade XI reduced to short internal branches, polytomies and low support values.

In recent years, plastomes of over 40 *Diospyros* species have been published (Fu *et al.*, 2016; Jo *et al.*, 2016; Turner *et al.*, 2016; Li *et al.*, 2018; Liu *et al.*, 2018; Huang *et al.*, 2023). These data constitute the foundation for a *Diospyros* plastid phylogeny yet are still restricted to economically important groups, such as *Diospyros kaki* (persimmon), along with its close relatives, or species restricted to certain geographic areas, such as New Caledonia and Temperate Asia. The broader relationships of most *Diospyros* species based on these extensive plastome data remain highly uncertain.

Southeast Asia is one of the hotspots of diversity for the genus (Fig. 1); however, the phylogenetic relationships of these species remain complex (Duangjai *et al.*, 2009; Turner *et al.*, 2013*a*). Sampling all ∼300 *Diospyros* species in Southeast Asia using field-collected samples alone to understand evolutionary relationships is a time- and budget-intensive prospect. To address these challenges, the use of DNA extracted from museum collections, e.g. herbarium specimens, via genome skimming has proven effective, providing DNA suitable for phylogenetic studies (Rowe *et al.*, 2011; Särkinen *et al.*, 2012; Staats *et al.*, 2013; Bakker *et al.*, 2016; Kates *et al.*, 2021). This use of herbarium specimens and genome skimming in the study of Asian *Diospyros* can bypass the challenges of fieldwork and exponentially increase the number of taxa available for inclusion in phylogenetic analyses. Current genome skimming approaches along with modern bioinformatics pipelines can turn legacy herbarium collections into essential current data to address such biodiversity knowledge darkspots. Additionally, genome skimming is currently the most used protocol for obtaining whole-plastid genomes for phylogenetic studies. In plants, whole plastomes have proven to be robust for phylogenetic reconstruction and successfully resolved ambiguous relationships in many closely related taxa (Parks *et al.*, 2009; Burke *et al.*, 2014, 2016; Eserman *et al.*, 2014; Givnish *et al.*, 2015; Guo *et al.*, 2017; Twyford and Ness, 2017; Bi *et al.*, 2018; Gitzendanner *et al.*, 2018; Ye *et al.*, 2018; Lloyd Evans *et al.*, 2019; Stettler *et al.*, 2021; Mo *et al.*, 2022; Yang *et al.*, 2022). Obtaining plastomes through genome skimming also offers maximum data yield when herbarium specimens are destructively sampled.

Although NGS has recently become commonly used in phylogenetic studies, it does not necessarily represent the most cost-effective short-term strategy for preliminary phylogeny building. Raw sequencing data from genome skimming typically contain only 0.5–20 % of the reads originated from the plastid genome, while the rest of the sequence data, consisting of nuclear and mitochondrial genomes, are not used during plastome assembly (Twyford and Ness, 2017). In scenarios in which funds for sequencing are limited, targeting the most informative plastid loci using Sanger sequencing may be a more cost-effective strategy to produce well-resolved topologies (Chen *et al.*, 2020; Foster *et al.*, 2021; Huang *et al.*, 2023). Combining Sanger sequence data from different sources is usually achieved by analysis of the largest common subset of data among the samples such that missing data are minimized (Roure *et al.*, 2013).

Lastly, previous phylogenetic studies of *Diospyros* based on Sanger sequencing have utilized plastid loci that have proven phylogenetically informative in other angiosperm studies (Duangjai *et al.*, 2006, 2009; Turner *et al.*, 2013*a*; Linan *et al.*, 2019). However, it was unknown whether these represent the most informative gene regions for *Diospyros.* A recent comparative plastome study of 45 *Diospyros* species representing five clades (out of 11 or 12 clades) by Huang *et al.* (2023) showed that the six plastid regions that are commonly used in *Diospyros* phylogenetic studies are not the most variable gene regions. Instead, they found that the most variable plastid sequences are located within the *rpl33*, *rpl22*, *petL*, *psaC* and *rps16* genes, along with several intergenic regions (Huang *et al.*, 2023). However, sampling was very limited, thus examining the variability of plastome regions across a more representative set of *Diospyros* species is crucial to identifying markers that can maximize phylogenetic signal where data are limited.

In this study, we sought to (1) assemble whole-plastid genomes for Asian *Diospyros* and reconstruct evolutionary relationships of *Diospyros*; (2) reconstruct a multi-locus chloroplast phylogeny based on legacy Sanger-sequence derived gene regions combined with the same regions mined from our novel plastome sequences to generate the most taxonomically complete phylogeny to date for the genus; and (3) identify the most informative plastid loci for generating a cost-effective genus-wide phylogeny for *Diospyros*.

## MATERIALS AND METHODS

### Plant material

Leaf tissue was obtained from a combination of field collections made between 2018 and 2022 as well as from herbarium specimens. Field-collected leaf material was dried in silica gel to preserve DNA at the highest quality (Chase and Hills, 1991; Särkinen *et al.*, 2012). Sampling from herbarium specimens focused on the collections held at the Royal Botanic Garden, Kew Herbarium (K), the University of Reading Herbarium (RNG) and the Oxford University Herbaria (OXF, FHO). About 2–4 cm^2^ of leaf sample (∼20–40 mg) was taken from each herbarium specimen and used for DNA extraction.

Sampling targeted primarily Southeast Asian *Diospyros* species along with species representing all the clades from other geographic areas inferred in the previous studies (Duangjai *et al.*, 2006, 2009; Turner *et al.*, 2013*a*; Linan *et al.*, 2019). *Euclea*, *Royena* and *Lissocarpa* were also sampled to be set as outgroups. Species identification of most herbarium samples was validated against type specimens or taxonomic literature. However, when identification could not be confidently confirmed, the original identification was retained for subsequent analyses or marked as ‘*conferatur* (*cf.*)’ to refer to the most morphologically similar species when available. In total, we sequenced 110 silica gel-dried and 214 herbarium samples representing around 186 identifiable taxa and 17 unidentified samples (Duangjai *et al.*, 2006, 2009; Turner *et al.*, 2013*a*; Linan *et al.*, 2019).

### Genomic DNA extraction, library preparation and DNA sequencing

Genomic DNA was extracted from leaf tissue using several methods, including a modified CTAB method (Doyle and Doyle, 1987), DNeasy Plant Mini Kit (Qiagen Ltd, UK) and DNeasy Plant Pro Kit (Qiagen Ltd, UK). The DNA extraction kits were only used for the silica gel-dried samples as they failed to extract enough DNA from herbarium specimens in preliminary experiments. For samples with high levels of secondary metabolites (usually indicated by a dark colour in the lysate solution), a sorbitol wash was applied to lysed samples to remove polysaccharides and polyphenols before CTAB extraction (Tel-zur *et al.*, 1999).

DNA extractions were quantified using a Qubit 3.0 fluorometer (Thermo Fisher Scientific, Inc., MA, USA), and qualified using NanoDrop microvolume spectrophotometers (Thermo Fisher Scientific, Inc., MA, USA) and gel electrophoresis using 0.7 % agarose gel. Generally, DNA samples that have ≥800 ng yield and A260/A280 between 1.7 and 2.1 were used to prepare NGS libraries. DNA samples that had lower quantity and purity were discarded except for when alternative samples for those species were not available.

DNA library preparation and whole-genome sequencing was carried out at Novogene (Cambridge, UK), the Jodrell Laboratory (Royal Botanic Gardens, Kew, UK), Macrogen (Seoul, Republic of Korea) and Arbor Biosciences (Ann Arbor, MI, USA). Of these, 69 DNA libraries were constructed in-house using the NEBNext Ultra II DNA Library Preparation Kit for Illumina (Ipswich, MA, USA) following a modified half-reaction protocol. DNA libraries were then captured and enriched using the Angiosperms353 probe set (Johnson *et al.*, 2019). DNA library molarity was estimated using the library yield measured by Qubit 3.0 fluorometer and the average library fragment size using TapeStation 4200 (Agilent, Santa Clara, CA, USA). In order to ensure plastid DNA fragments were sufficiently sequenced, the pre-captured DNA libraries (for high-copy genomes including the plastid genome) were spiked into the nuclear enriched libraries at the ratio of around 30:70 by mass (calculated using molarity). DNA libraries were sequenced using either Illumina Novaseq 6000 or HiSeq X sequencing platforms, with 150-bp paired-end reads (PE150), generating ∼1 Gbp per sample. In addition to samples prepared in-house, we utilized whole-genome skimming data for an additional 96 samples from our collaborators at Singapore Botanic Gardens and the Royal Botanic Garden Edinburgh, all using the PE150 Illumina sequencing system. In total, this study included sequencing data for 364 samples prior to the read quality control steps.

### Plastome assembly and annotation

Raw reads were trimmed to remove adaptors and low-quality reads using TrimGalore! v.0.6.5 (Krueger, 2019) with minimum Phred score of 30 and minimum length of 20 bp. Plastomes were assembled *de novo* using GetOrganelle v.1.7.6.1 (Jin *et al.*, 2020), Fast-Plast v.1.2.9 (McKain and Wilson, 2020) and NOVOPlasty v.4.3.1 (Dierckxsens et al., 2017). These three plastome assembly tools were chosen because they have been proven to have the highest success in plastome assembly and accuracy compared with other plastome assemblers (Freudenthal *et al.*, 2020). In addition to the default parameters of these assembly tools, we used the built-in Ericales Bowtie index when running Fast-Plast. For NOVOPlasty, the plastome of *Diospyros filipendula* (AB925317) was used as the seed. For GetOrganelle, the command ‘k 21,45,65,85,105’ was supplied for running assembly in SPAdes (Bankevich *et al.*, 2012). The coverage cut-off value was automatically calculated in SPAdes. When samples failed to produce complete plastomes, these samples were reassembled using complete plastomes of closely related species generated from the previous step as references (using the flag ‘-s’ in GetOrganelle) for read mapping; however, this step only slightly improved the results. The assembly summary was calculated using the command ‘summary_get_organelle_output.py’ in GetOrganelle.

Only plastomes that could be successfully assembled into a complete circular genome were annotated. These plastomes were automatically annotated using GeSeq (Tillich *et al.*, 2017) with annotation support by Chloë Annotator (Zhong, 2020) (hereafter referred as GeSeq + Chloë). Additionally, annotation of a few plastomes was carried out manually by transferring the annotations of *Nicotiana tabacum* (Z00044.2) (Shinozaki *et al.*, 1986) and *Diospyros kaki* (KT223565) (Fu *et al.*, 2016) to our plastomes using Geneious Prime v.2022.2.1 (www.geneious.com). The start and stop codons of each exon were manually adjusted against the open reading frames (ORFs) using Geneious Prime. Following both annotation methods, plastomes were compared to ensure that no major conflicts were found. Considering their automation and reproducibility, we used GeSeq + Chloë as a standard protocol to annotate the rest of the plastomes, which were then used in downstream analyses and submitted to GenBank.

Additionally, 50 published *Diospyros* plastomes were downloaded from GenBank (Clark *et al.*, 2016; Fu *et al.*, 2016; Jo *et al.*, 2016; Turner *et al.*, 2016; Li *et al.*, 2018; Liu *et al.*, 2018) (Supplementary Data Table S2). These plastomes were re-annotated using GeSeq + Chloë to ensure compatibility with the newly generated plastomes. Further inspection and editing were done manually when necessary using Geneious Prime. All protein-coding, tRNA and rRNA genes were extracted for the plastid gene analyses.

Finally, in order to determine whether any large rearrangements of portions of the plastome occurred among *Diospyros* species, nine whole plastomes of *Diospyros* representing all the nine clades resolved in the phylogenetic analysis of this study, one *Euclea* plastome and one *Royena* plastome were aligned and compared with the plastome of *N. tabacum* (Z00044.2) using the progressiveMauve algorithm (Darling *et al.*, 2010).

### Six-locus assembly

In order to maximally expand the taxonomic sampling breadth of *Diospyros*, the most commonly available six plastid loci (*atpB*, *ndhF*, *matK*, *rbcL*, *trnL–trnF* and *trnS–trnG*) based on previously published Sanger sequence data were also assembled. This step allows us to include samples that failed the whole plastome assembly but may contain sufficient usable reads from these regions. The adaptor- and quality-trimmed reads were mapped to the reference six markers extracted from the plastome of *Diospyros ferrea* (K-DFER-030) using Bowtie 2 v.2.4.5 (Langmead and Salzberg, 2012) with the command ‘–local –score-min G,20,8’. Then, mapped reads (.sam) were sorted and converted to binary format (.bam) using SAMtools v.1.18 (Li *et al.*, 2009). Genotype likelihoods were generated using the command ‘mpileup -E’ in BCFtools (Li, 2011*b*) with base alignment quality (BAQ) recalculated using a profile hidden Markov model (HMM) for more accurate SNP calling (Li, 2011*a*). This step produced variant call files (.vcf) that were then converted into nucleotide sequences (.fasta) using Samtools. Uncertain sequences were marked as ‘N’. These gene files were annotated and inspected using Geneious Prime before data submission and downstream analysis.

### Datasets and alignments

**Fig. 2.**
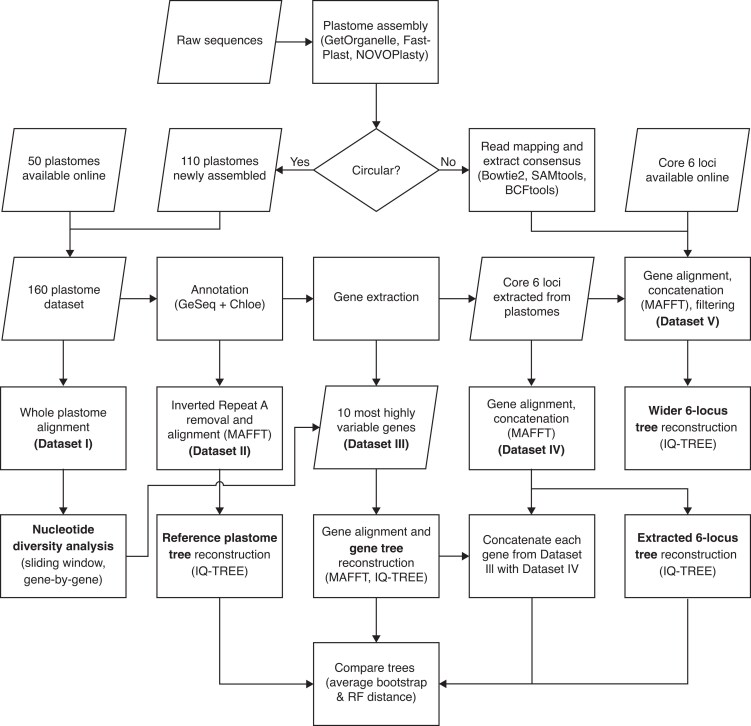
A schematic workflow showing how each dataset was generated and analysed. Parallelogram boxes (▱) represent input or data; rectangle boxes (▭) represent process; diamond boxes (◇) represent decision check point.

Data were divided into five datasets using multiple different alignment schemes and each was used for a separate analysis (described in Table 1 and Fig. 2). All DNA alignments were made using MAFFT v.7.515 (Katoh and Standley, 2013) with the command ‘auto’.

**Table 1.** A summary of the alignment and best-fit substitution models of the datasets used in this study. The core six loci mentioned in Dataset IV are *atpB*, *trnK–matK*, *trnL–trnF*, *trnS–trnG*, *ndhF* and *rbcL*. Please refer to the IQ-TREE manual for details on substitution models.

Dataset	Locus/region	Number of samples	Alignment length (bp)	Number and percentage of variable sites	Undetermined characters (−, ?, *N*)	Best-fit substitution model
I	Whole plastome (with both IRs)	160	176 307	24 884 (14.1 %)	3 008 667 (10.67 %)	No phylogenetic reconstruction
II	Plastome (with one IR)	160	149 491	24 039 (16.1 %)	2 892 302 (12.09 %)	TVM + F + I + I + R2
III	*ycf1*	160	5898	1488 (25.2 %)	48 395 (5.12 %)	TVM + F + I + R3
	*rpoC2*	160	4311	647 (15.0 %)	23 940 (3.47 %)	TVM + F + I + I + R2
	*trnK–*UUU (including *matK*)	160	2749	607 (22.1 %)	27 875 (6.34 %)	TVM + F + I + I + R3
	*ndhF*	160	2460	529 (21.5 %)	32 871 (8.35 %)	TVM + F + I + I + R2
	*ndhA*	160	2500	493 (19.7 %)	44 859 (11.22 %)	TVM + F + I + I + R2
	*rpoC1*	160	2884	435 (15.1 %)	11 966 (2.59 %)	K3Pu + F + R2
	*rpoB*	160	3216	423 (13.2 %)	1038 (0.20 %)	TVM + F + R2
	*clpP1*	160	2415	396 (16.3 %)	57 042 (14.70 %)	K3Pu + F + I + I + R2
	*matK*	160	1539	351 (22.8 %)	4380 (1.78 %)	TVM + F + R2
	*rpl16*	160	1633	343 (21.1 %)	39 207 (15.08 %)	K3Pu + F + I + I + R2
IV	Core six-locus (extracted from the 160 plastomes)	160	8359	1552 (18.6 %)	118 328 (8.47 %)	TVM + F + I + I + R3
	Core six-locus + *ycf1*	160	14 257	3040 (21.3 %)	161 613 (7.01 %)	TVM + F + I + R4
	Core six-locus + *rpoC2*	160	12 670	2199 (17.4 %)	137 158 (6.77 %)	TVM + F + I + R4
	Core six-locus + *ndhA*	160	10 859	2044 (18.8 %)	158 077 (9.10 %)	TVM + F + I + I + R3
	Core six-locus + *rpoC1*	160	11 243	1987 (17.7 %)	125 184 (6.96 %)	TVM + F + R3
	Core six-locus + *rpoB*	160	11 575	1975 (17.1 %)	114 256 (6.17 %)	TVM + F + I + R4
	Core six-locus + clpP1	160	10 785	1948 (18.1 %)	170 260 (9.87 %)	TVM + F + I + R3
	Core six-locus + *rpl16*	160	9958	1895 (19.0 %)	148 127 (9.30 %)	TVM + F + I + I + R3
V	Wider six-locus	527	9647	2487 (25.8 %)	1 130 855 (26.25 %)	TVM + F + I + I + R4

### Nucleotide diversity analysis

To identify the most variable regions of the plastome of *Diospyros*, we (1) calculated nucleotide diversity using a sliding window approach (*π*; Nei and Li, 1979) and (2) conducted a gene-by-gene variable site assessment across the whole-plastome alignment.

#### Nucleotide diversity

This was calculated across the whole-plastome alignment of 160 Ebenaceae samples (Dataset I, 114 taxa, 176 307 bp) in DnaSP v.5 (Librado and Rozas, 2009) using the sliding window approach with window sizes of 200, 600 and 1000 bp and step size of 50 bp. Alignment columns that contained at least 50 % gaps were removed using Geneious Prime before calculating the nucleotide diversity. This metric was chosen as it measures the average number of nucleotide differences per site within any random pairs of homologous sequences (Nei and Li, 1979). To estimate gene position across this alignment, the majority consensus sequence was extracted and annotated using Geneious Prime based on sequence homology at 50 % similarity threshold. Then, the nucleotide diversity windows across the entire annotated alignment were examined. This enabled us to identify regions with high and low nucleotide diversity along the plastomes, including the intergenic spacers.

#### Gene-by-gene comparison

In addition to examining nucleotide diversity across the plastome, we also conducted a gene-by-gene comparison by examining matrices of variable sites of each gene extracted from the gene annotations of the 160 plastomes in Dataset I. Instead of measuring average genetic distance within a portion of alignment, the number of variable sites were directly counted from any sites in an alignment where at least two different types of nucleotide occur, and the proportion of variable sites to the length of each gene alignment was calculated (Höglund, 2009). The implementation of this metric is straightforward and effective when comparing multiple gene alignments with different lengths as gene boundaries are better defined and consistent than using sliding windows. The number and proportion of variable sites of each gene alignments were calculated by AMAS v.1.0 (Borowiec, 2016). In general, genes containing intron(s) were treated as a single unit in the analysis. Due to the widespread use of *matK* in systematics, this gene was also analysed separately despite being embedded within the *trnL* intron. Unlike coding sequences, intergenic spacers were not included in this analysis as their alignment homology can be error-prone due to the common occurrence of indels, such as *trnH–psbA*, which has been found difficult to align even among closely related taxa (Chase *et al.*, 2007), and possible gene rearrangement in distantly related taxa (Kelchner, 2000).

### Phylogenetic analyses

#### Reconstruction of the reference plastome tree (Dataset II)

We first created a plastome phylogeny (Dataset II) by removing one of the inverted repeats (IRs) from plastomes in Dataset I, representing 114 Ebenaceae taxa (109 *Diospyros* taxa along with two *Euclea* and three *Royena* species as outgroups) for which whole-plastome data are available. This tree is used as the reference phylogeny for the topology comparison with other trees generated from reduced datasets described in the following sections (see Table 1).

Following the alignment step using the same method as in the nucleotide diversity analysis, phylogenetic trees were reconstructed under the maximum likelihood inference in IQ-TREE v.2.2.0.3 (Nguyen *et al.*, 2015; Minh *et al.*, 2020) with 1000 ultrafast bootstrap replicates generated by the built-in UFBoot2 (Hoang *et al.*, 2018). The nucleotide substitution models were calculated using the built-in ModelFinder (Kalyaanamoorthy *et al.*, 2017) in IQ-TREE. The phylogenetic tree analysis was run on the UK’s Crop Diversity Bioinformatic HPC (The John Hutton Institute and the National Institute of Agricultural Botany). The phylogenetic trees were initially inspected and rooted using the clade containing *Euclea* and *Royena*. Then, the rooted tree files were visualized via the R packages ggtree v.3.10.0 (Yu et al., 2017), treeio v.1.26.0 (Wang *et al.*, 2020), tidyverse v.2.0.0 (Wickham *et al.*, 2019) and ggplot2 v.3.4.4 (Wickham, 2009) in RStudio v.2023.09.1 (RStudio Team, 2020). Tree tips were coloured according to the biogeographic regions where the species naturally occur (Olson *et al.*, 2001).

#### Reconstruction of the highly variable plastid gene trees (Dataset III)

Although nucleotide diversity calculated using *π* with sliding windows can show us the regions across the whole plastome that have high nucleotide variations, it is not practical to extract these regions for downstream analysis as their boundaries (a sliding window) are not likely to be well defined across different analyses, which in turn can result in a less reproducible workflow. Therefore, to perform individual plastid gene tree analysis we ranked all plastid genes using the results from the gene-by-gene variable site analysis. Then, the ten most variable plastid loci were extracted from the 160 whole plastomes (Dataset III) and individually aligned using MAFFT as described above. It is important to note that some loci that resulted in a high proportion of variable sites are relatively short and do not provide sufficient phylogenetic signal. As a result, we decided to include gene alignments that have the minimum length of 200 bp in this analysis. Each gene tree was reconstructed using IQ-TREE following the methods described above. The clade containing *Euclea* and *Royena* was set as the outgroup.

#### Reconstruction of the multi-locus phylogenetic trees (Dataset IV)

To integrate our data with existing DNA sequencing data of *Diospyros* (Duangjai *et al.*, 2009; Turner *et al.*, 2013*a*; Linan *et al.*, 2019), we created Dataset IV, which comprises the six commonly available plastid markers of Ebenaceae: *atpB*, *ndhF*, *rbcL*, *trnK* intron (including *matK*), *trnL–trnF* (including *trnL* intron and *trnL–trnF* spacer) and *trnS–trnG* spacer (Duangjai *et al.*, 2006; Duangjai *et al.*, 2009; Turner *et al.*, 2013*a*; Linan *et al.*, 2019).

Dataset IV was generated by directly extracting these six markers from the 160 plastomes. Then, each extracted gene was aligned using the same method as above. Alignments were concatenated before the phylogenetic inference step using the same IQ-TREE pipeline as previous analyses.

In addition to the most phylogenetically informative loci identified in Dataset III, we evaluated which of these loci can maximize the phylogenetic resolution of the core six loci. We combined each of the ten genes with the highest number of variable sites with the core six loci to create subsets of Dataset IV. These alignments were used for the phylogenetic tree reconstruction.

#### Reconstruction of the wider six-locus tree (Dataset V)

Dataset V (the wider six-locus tree) included the DNA sequences of the core six loci from Dataset IV and two additional sources: (1) direct downloads of the six markers from GenBank, and (2) gene assemblies produced from the raw reads of the samples that failed whole-plastome assembly.

The DNA alignments were made using the same algorithm as the other datasets and inspected in Geneious Prime. Samples that appeared noticeably misaligned were manually removed. Note that all the samples that were removed appeared to be public data downloaded from GenBank. The remaining sequences were re-aligned. Then, we used trimAl v.1.4.1 (Capella-Gutierrez *et al.*, 2009) and the algorithm ‘-automated1’ to remove poorly aligned regions and used the command ‘pxclsq -p 0.5 -t’ in Phyx v.1.1 (Brown *et al.*, 2017) to remove samples that have nucleotide occupancy <50 % of the whole alignment. Then, all six alignments were concatenated, and finally samples with <50 % total nucleotide occupancy in the concatenated alignments were removed. After these trimming steps, the final alignment of the wider six-locus dataset (Dataset V) contained 527 samples of around 250 *Diospyros* and 13 outgroup taxa (Supplementary Data Table S2). The tree was rooted using the *Lissocarpa* clade as Lissocarpoideae is sister to Ebenoideae (Duangjai *et al.*, 2006).

#### Phylogenetic tree comparison

Following the generation of phylogenies described in the previous sections, we compared the similarity of these trees with the reference plastome tree by (1) visualizing the clade topologies between trees (tanglegrams), (2) calculating the average branch bootstrap support across each tree, and (3) using the weighed Robinson–Foulds distance (wRF; Robinson and Foulds, 1981). The latter metric compares the number of clade splits that are unique to one of the trees. The wRF distances of genes were calculated against the reference plastome tree using the command ‘wRF.dist’ in the R package phangorn v.2.11.1 (Schliep, 2011).

## RESULTS

### DNA extraction and library preparation

Silica-gel-preserved samples yielded an average of 792.74 ng of DNA (range 0–8390.4 ng, s.d. ± 984.50), whereas herbarium samples yielded an average of 380.83 ng (range 0–3480 ng, s.d. ± 563.81) (Table 2). All *Lissocarpa* samples, except one, failed to yield sufficient DNA for library preparation and sequencing. Samples with low to medium DNA yield (60–800 ng DNA, 30 samples from 18 species of silica-preserved material and 39 samples from 31 species of herbarium material) were carefully processed using in-house library preparation. Samples with higher yield (63 samples from 42 species of silica-dried material and 173 samples from 124 species of herbarium material) mostly had library preparation outsourced. One hundred and fifty-five DNA extracts from newly collected silica-preserved samples (38 species) and 491 herbarium extractions (211 species) were excluded from library preparation as they had insufficient DNA quantity or low quality, or simply represented non-target species or replicates of species already sampled (Table 2). All remaining DNA extracts and libraries were archived at −80 °C at the School of Biological Sciences, University of Reading, UK.

**Table 2.** DNA extraction yields used for NGS library preparation and sequencing, grouped by sample type (herbarium specimens vs newly collected, silica gel-preserved material) and by library preparation facility. For each category, the minimum, maximum, mean and standard deviation of DNA input (ng) are reported, along with the number of species and total samples (including those excluded from library preparation and sequencing). Excluded taxa include those with very low DNA yields and duplicate samples that were not needed for this study.

Sample type	Library preparation facility	Minimum DNA (ng)	Maximum DNA (ng)	Mean DNA (ng)	Standard deviation	Number of species	Number of samples (*n*)
Herbarium	In-house	120.75	306	208.63	43.77	31	39
	Outsourced	132	3230	776.82	534.67	124	173
	Excluded	0	3480	254.98	530.4	211	491
Newly collected	In-house	68.64	759.2	280.63	143.88	18	30
	Outsourced	173	3170	1044.33	552.98	42	63
	Excluded	0	8390.4	789.6	1162.13	38	155

### DNA sequencing

The number of paired-end reads ranged from 154 043 to 26 970 979 (average 6 808 882.42). DNA sequencing data that were originated from the same original leaf samples were merged before the subsequent analysis. In total, the final set of newly generated read data comprised 324 samples of ∼186 identifiable taxa and 17 samples with ambiguous species identification (Supplementary Data Table S1).

### Plastomes of *Diospyros*, *Euclea* and *Royena*

In total, 110 circular plastomes were assembled (62 from silica gel-dried and 48 from herbarium samples), while 213 samples (165 herbarium and 49 silica gel-dried) failed to yield complete circular plastomes. Of the three plastome assembly tools used in this study, GetOrganelle had the highest success in plastome assembly (110) followed by Fast-Plast (81) and NOVOPlasty (73). The assembly summary showed that the plastome of *Diospyros cathayensis* (K-04-003) has the lowest average base coverage of 30.4 while *D. lanceifolia* (K-6B-050) has the circular plastome with the highest average base coverage of 589.9; both are herbarium samples. Among samples yielding fully circularized plastomes, the lowest DNA inputs were 109.72 ng for a silica gel-dried specimen (*D. tahanensis*, NM214) and 258.0 ng for a herbarium specimen (*D. littorea*, K-6E-098, 48 years old); sequencing libraries of both samples were prepared in-house.

**Fig. 3.**
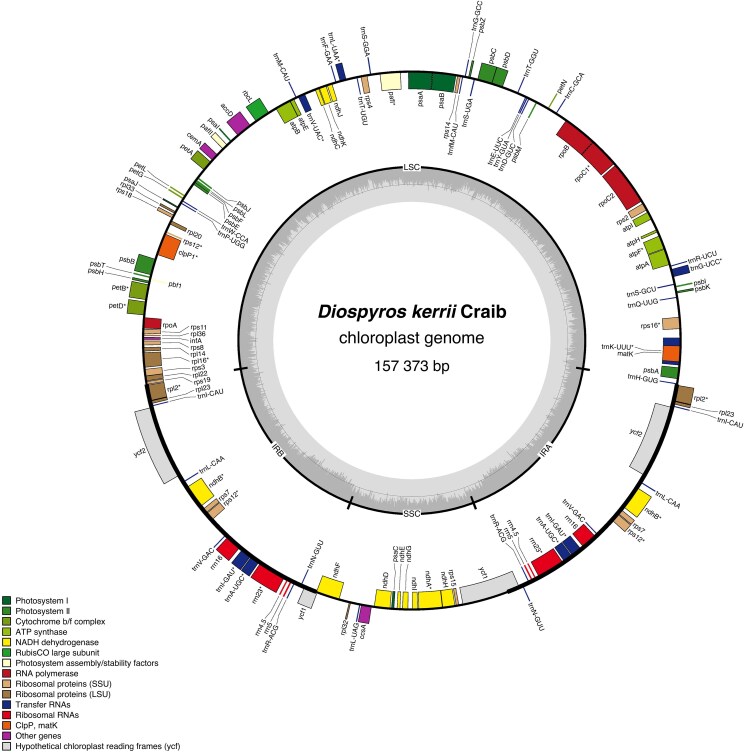
The whole plastome of *Diospyros kerrii*. This annotated plastome was drawn in OGDRAW (Greiner *et al.*, 2019) showing its standard quadripartite structure, and genes are coloured by their functions.

**Fig. 4.**
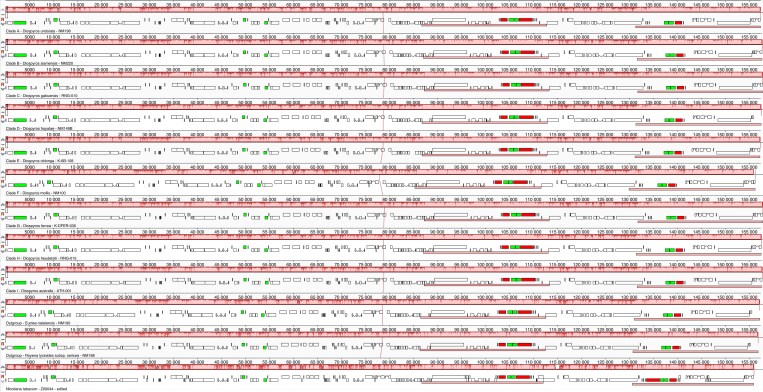
Comparative plastome alignments of representative plastomes all nine *Diospyros* clades, *Euclea*, *Royena* and *Nicotiana tabacum* (from top to bottom) using progressiveMauve (Darling *et al.*, 2010). The white blocks are protein-coding genes, the green blocks are tRNA genes and the red blocks are rRNA genes. The vertical red line around the middle of the figure indicates the connections between homologous blocks across the samples. In this case, there is only one single block and there is no evidence of gene relocation.

All plastomes of *Diospyros*, *Euclea* and *Royena* have a quadripartite structure with a size between 156 214 and 158 201 bp (Fig. 3). None of the *Lissocarpa* plastomes could be circularized successfully in our study. The plastomes of all samples comprise 113 genes, categorized by function into 79 protein-coding genes (CDSs), 30 tRNA genes, and 4 rRNA genes. Of these, six CDSs and seven tRNA genes, and all rRNA genes are located within the IRs. Additionally, the genes *ndhD* and *rpl2* were not automatically annotated using GeSeq + Chloe in 4 and 40 plastomes, respectively. These genes were manually annotated using Geneious Prime (Supplementary Data Table S1). Additionally, the boundary of *ycf1* was manually edited for two plastomes. The GC content consistently ranged between 37.3 and 37.5 %. The progressiveMauve analysis of *Diospyros* plastomes from all the clades resolved in the phylogenetic analysis along with the reference *N. tabacum* plastome did not detect any substantial rearrangements (Fig. 4).

### Nucleotide diversity

**Fig. 5.**
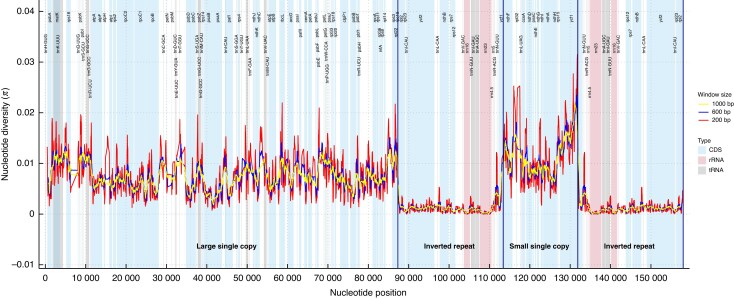
Nucleotide diversity (*π*) of the plastome alignment (Dataset I) using the sliding window approach implemented in DnaSP v.5 with window size 200, 600 and 1000 bp and step size 50 bp. The vertical colour bands indicate the types of plastid genes while the white spaces show non-coding regions of the plastid genome.

The alignment of whole plastomes of *Diospyros*, *Euclea* and *Royena* showed that the small single-copy region (SSC) harbours the highest nucleotide diversity (*π*), followed by the large single-copy region (LSC), whereas the IRs are more conserved among taxa. When considering nucleotide diversity along the whole alignment using the sliding window method, the results from every analysis using three different window sizes (200, 600 and 1000 bp) showed that the region within SSC towards the 5′ end of *ycf1* is the most diverse (Fig. 5). Moreover, non-coding spacers typically showed high nucleotide diversity, most prominently when using the shortest sliding window size (200 bp). Despite the window size, the intergenic spacers located near the beginning of the SSC, namely, *ndhF–rpl32* and *rpl32–trnL* (UGA), and portions of the genes *rbcL*, *rpl22* and *ycf1*, are also hotspots of nucleotide mutations.

**Fig. 6.**
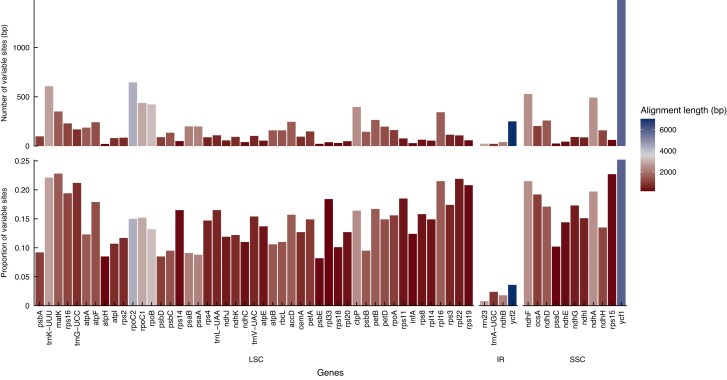
Number and proportion of variable sites per gene alignment. Genes are arranged and sorted into the order they present within Ebenaceae plastomes. The bar colour gradient represents alignment lengths. Any alignment <200 bp long and with <20 variable sites is not shown here.

Similar patterns were observed in the number of variable sites across all the plastid genes (Fig. 6). The gene *ycf1* in the SSC harbours the most variable sites (1483 bp), followed by *rpoC2*, *trnK* (UUU), *ndhF*, *ndhA*, *rpoC1*, *rpoB*, *clpP1*, *matK* and *rpl16* (Table 1, Supplementary data Table S3). Among these genes, *ycf1* also has the highest proportion of variable sites (25.2 %), followed by *matK*, *trnK*, *ndhF* and *rpl16*, which contain at least 20 % variable sites, while the other genes have lower proportions of variable sites. The longest plastid gene present in Ebenaceae, *ycf2*, (6813–6903 bp) has 3.6 % (119 bp) of variable sites from the alignment of these 160 samples. Most plastid genes in the LSC and SSC were found to have a higher proportion of variable sites than those genes within the IRs.

### Phylogenetic analysis

Our reference whole-plastome tree (Dataset II) representing 160 samples was compared with phylogenetic trees reconstructed from subsets of genes, including the most variable genes identified (Dataset III), and the most commonly used six loci with the addition of highly variable genes (Dataset IV). Lastly, the expansive phylogenetic tree using all available sequencing data of the core six loci (Dataset V), containing over 500 samples, was also reconstructed and represents the most comprehensive phylogeny of Ebenaceae to date (Supplementary Data Table S2).

#### Reference plastome tree (Dataset II)

**Fig. 7.**
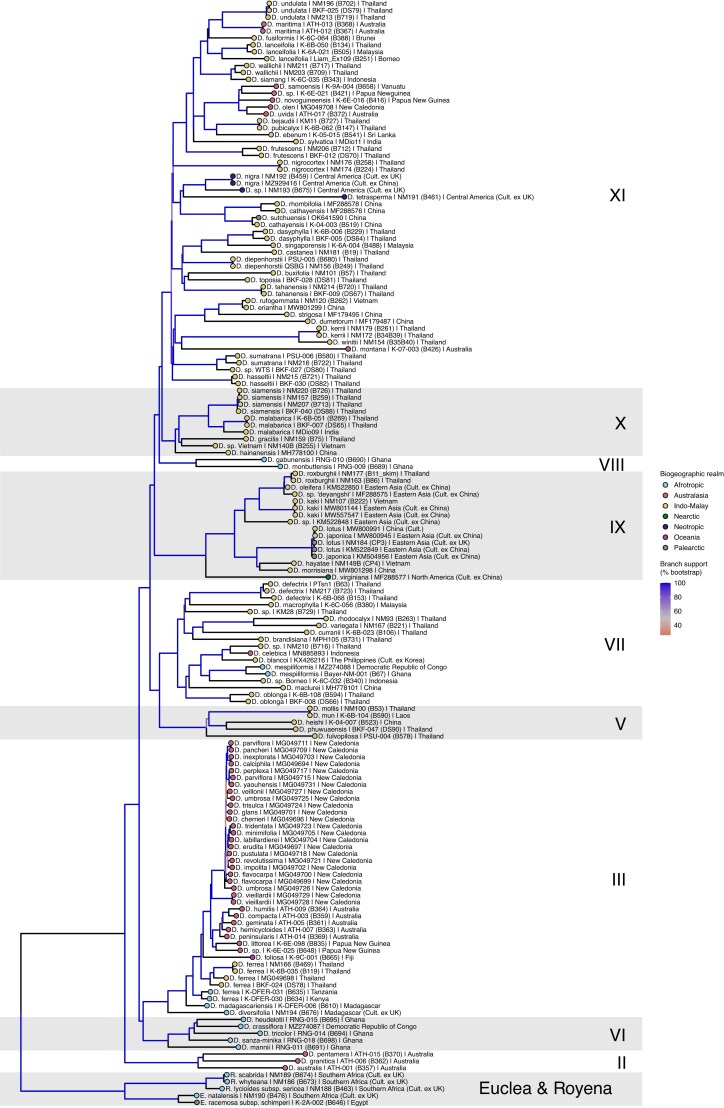
Estimated maximum likelihood phylogeny inferred from the whole-plastome alignment (Dataset II) of 160 Ebenaceae samples (without *Lissocarpa*). The branch bootstrap support (1000× UFBoot) is shown in a colour scale. The branch lengths are relative to the number of nucleotide substitutions per site (the scale is not shown here). See Supplementary data all_trees.trees’ for the original tree files with bootstrap and branch length annotations. Tips of the tree are coloured by the biogeographic realms (Olson *et al.*, 2001).

The plastome tree (160 tips, 114 taxa) was well supported, with all nine major clades showing high bootstrap support values (BS 98–100) (Fig. 7). However, the placements of some minor clades within Clades III and XI are uncertain, with lower bootstrap support (BS <80 %) and short branch lengths. Southeast Asian samples are found in nearly all clades except Clades II, IV and VIII. Additionally, in the plastome tree with other Ericales species as outgroups (*Ardisia polysticta*, *Pouteria altissima*, *Primula cicutariifolia* and *Tieghemella africana*), *Euclea* and *Royena* are resolved sister clades, and *Diospyros* is sister to the clade containing both clades (this tree is not present here). These species were not used as outgroups in the final reference plastome tree due to their high divergence from the rest of Ebenaceae, resulting in long internal branches.

#### Highly variable plastid gene trees (Dataset III)

Gene trees produced from the ten most variable gene alignments in Dataset III (Table 1) individually did not provide high bootstrap support (average BS <80 % across trees; see Supplementary Data Table S4) and tended to generate short branch lengths and internodes, with the exception of the *ycf1* gene. The *ycf1* gene tree is comparatively well resolved and shows the highest similarity to Dataset II (wRF 0.154) and 83 % average bootstrap support across the tree, while other gene trees have lower similarity scores and lower average bootstrap support.

#### Multi-locus trees with addition of highly variable genes (Dataset IV)

**Fig. 8.**
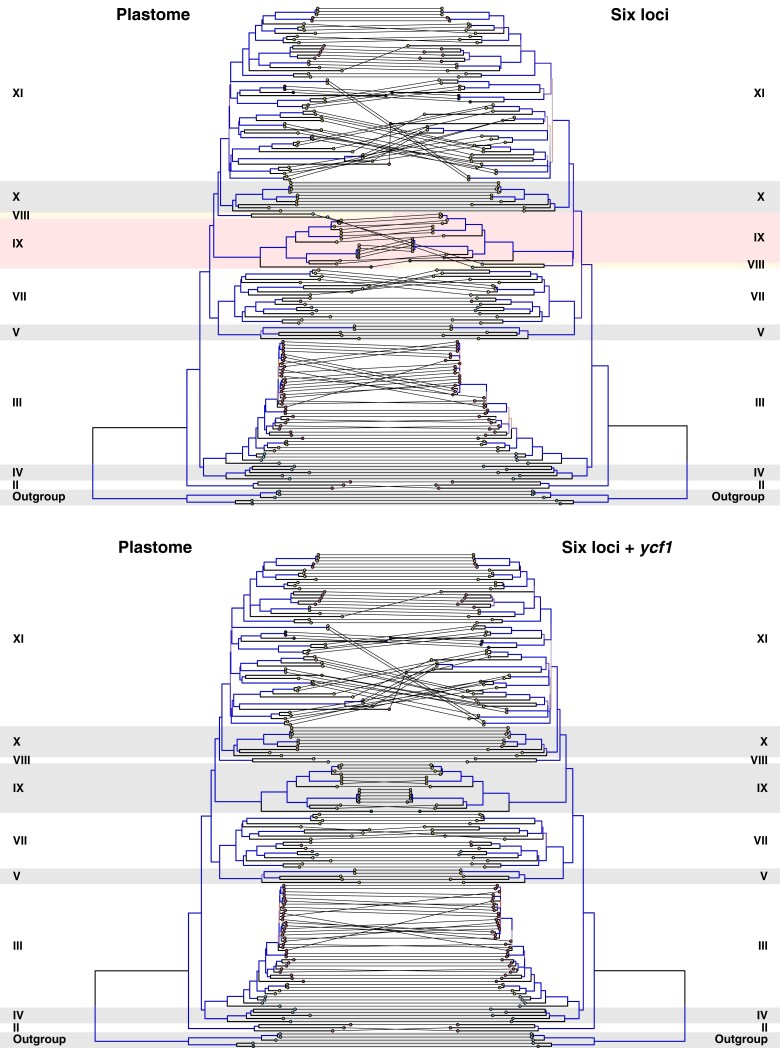
Comparison of phylogenetic trees inferred from the reference plastome dataset (Dataset II; upper and lower left), the core six-locus dataset (Dataset IV; upper right), and the six-locus dataset with the addition of *ycf1* (lower right). Differences in clade placement among trees are highlighted in yellow and red.

The comparison between trees generated from the six core loci (Dataset IV) and our reference plastome phylogeny (Dataset II) displayed similar topologies (Fig. 8). However, the placement of Clades VIII, IX and X differs between the phylogenetic trees arising from the core six plastid markers and these six markers with an addition of the following highly variable genes: *clpP1*, *ndhA*, *rpl16*, *rpoC1* and *rpoC1.* Within these trees, Clade IX is resolved as sister to Clade VIII with short internodes (BS 82, 89, 84, 97 and 84 %, respectively). On the other hand, Clade IX is resolved as sister to Clades VIII + X + XI in Dataset II and the six-locus with *rpoB* or *ycf1* phylogenies (BS 99 and 100 %, respectively; Fig. 8). In addition, the bootstrap support along the backbone of Clade XI of all the trees in Dataset IV is lower (BS 30–50 %) compared with the same nodes on the tree derived from Dataset II (BS >70 %).

Of the topologies arising from the core six-locus alignment with each of the highly variable genes, we found that only two trees had average bootstrap values exceeding 90 % arising from the core six loci + *ycf1* and six loci + *rpoC2* alignments. Moreover, the topology of the six-locus + *ycf1* phylogeny is resolved with the lowest wRF distance of all topology comparisons made between various genes and the reference plastome tree (Table 3, Fig. 8).

**Table 3.** Comparison between phylogenetic trees generated from different datasets in this study. The matrices used here are average BS across all splits and wRF distance to the reference plastome tree (Dataset II). Note that Dataset I was used for the nucleotide diversity analysis only while the wider six-locus tree (Dataset V) is not shown here as it contains a different set of taxa and is not suitable for wRF distance interpretation.

Dataset	Detail	Average bootstrap support	Weighted Robinson–Foulds distance (wRF) to the reference tree
II	Plastome (with one IR)	95.40	Reference tree
III	*ycf1*	83.46	0.15461973
	*rpoC2*	78.03	0.19703861
	*trnK-UUU* (including *matK*)	76.26	0.17322232
	*ndhF*	76.72	0.17358439
	*ndhA*	72.52	0.18880468
	*rpoC1*	69.10	0.22423714
	*rpoB*	62.59	0.25045024
	*clpP1*	66.65	0.21829094
	*matK*	60.89	0.20547674
	*rpl16*	70.25	0.24919992
IV	Six-locus (160 tips)	87.63	0.10422391
	Six-locus + *ycf1*	90.63	0.08602638
	Six-locus + *rpoC2*	90.89	0.10652159
	Six-locus + *ndhA*	89.12	0.08957672
	Six-locus + *rpoC1*	87.26	0.10843245
	Six-locus + *rpoB*	87.26	0.11340084
	Six-locus + *clpP1*	89.13	0.09392429
	Six-locus + *rpl16*	88.15	0.09723179

#### The wider six-locus tree (Dataset V)

The ingroup clades of the wider six-locus phylogeny with around 263 Ebenaceae taxa (250 *Diospyros* taxa) (527 samples) (Dataset V) were generally well supported except for Clades III and XI, where there are multiple internal branches with very short internodes and there is low bootstrap support (BS <80 %). Using *Lissocarpa* as the outgroups, *Euclea* and *Royena* are well resolved as sister clades, which together form a sister clade to *Diospyros*. Although Clades III and XI both have low phylogenetic resolution, these clade placements occur in different patterns. In Clade III there are poorly supported placements of many taxa, indicated by short branch lengths and low bootstrap support along the backbone and towards the tips. In Clade XI, while the relationships near the tips of the branches are resolved, the deeper branching patterns have short internodes, lacking both resolution and support.

Other clade splits are similar to Dataset II, with the exception of Clades VIII and IX being sister clades in this phylogeny while Clade IX is resolved as sister to Clades VIII + X + XI in Dataset II (Tree S1). Clade I, comprising *D. maingayi* and *D. puncticulosa*, is resolved as a sister to the rest of *Diospyros*. These two species were not included in Datasets II–IV.

## DISCUSSION

We reconstructed phylogenetic trees including more than 100 species of *Diospyros* using whole-plastid genomes (Dataset II), which resolved the majority of previously unresolved relationships, especially the placement of clades VIII, IX and X. The multi-locus dataset that incorporated published Sanger sequence data (Dataset V) allowed the generation of the most taxonomically comprehensive phylogeny to date for the genus, resolving relationships of over 250 *Diospyros* species. With whole-plastome data of 114 Ebenaceae taxa, representing around 109 *Diospyros*, 3 *Euclea* and 2 *Royena* taxa, we identified that *ndhF–rpl32* and *rpl32–trnL* (UAG) intergenic spacers and portions of the *ycf1* gene contain the highest nucleotide sequence diversity. Of the six regions that are commonly used in *Diospyros* phylogenetic studies (*atpB*, *trnK–matK*, *trnL–trnF*, *trnS–trnG*, *ndhF* and *rbcL*), *trnK*, *matK* and *ndhF* have been found to be among the most variable while *atpB* and *rbcL* are less so. The resolution of the trees arising from the six regions combined is greatly improved by the addition of further highly variable plastid genes; in particular, *ycf1* gave similar resolution to the whole-plastome phylogeny.

Data from the whole-plastid genomes of *Diospyros* and other Ebenaceae were used in our study for nucleotide diversity and phylogenetic analyses. We maximized the number of plastid genomes used by extracting DNA from newly collected samples preserved in silica gel and from herbarium specimens. Three plastome assembly tools were used to increase success of the assemblies and ensure assembly accuracy. GetOrganelle demonstrated the highest success in plastome assembly for DNA extracted both from silica gel-dried and herbarium samples. We were able to assemble circularized plastomes of 110 samples (out of 324), 56 % of the silica-dried samples and 23 % of the herbarium samples. Our results concur with Bakker *et al.* (2016), who found that there were proportionally fewer plastid reads in genome-skimming data from herbarium specimens than from silica samples and generally showed only slightly shorter post-assembly contigs. Furthermore, achieving complete assembly of the plastome could become more challenging when working with DNA extracted from herbarium specimens due to various factors, such as badly preserved samples, highly degraded DNA input and low-quality sequencing reads (e.g. stemming from low complexity, short-length DNA libraries and adaptor contamination) (Staats *et al.*, 2011; Dabney *et al.*, 2013; Kistler *et al.*, 2017). One of the most prevalent factors potentially damaging herbarium DNA, particularly from tropical regions, is the historic treatment with alcohol, formaldehyde and mercuric chloride solution (Hodge, 1947; Forrest *et al.*, 2019).

### Recommendations for the use of herbarium specimens as a DNA source

In this study, we used strict quality control thresholds to decide whether DNA samples should be sequenced. For example, the general minimum cut-off for DNA yield for the outsourced library preparation was ≥800 ng with acceptable A260/280 absorbance of 1.7–2.1. However, Kates *et al.* (2021) found that there is no strong correlation between DNA yield and success in sequencing of on-target DNA fragments, suggesting this control could be relaxed. We also demonstrated that many samples with DNA input as low as around 150–200 ng also achieved circular plastomes when we carefully developed sequencing libraries in-house. The use of qPCR to quantify relative plastid genome content (Lee *et al.*, 2006; Harshitha and Arunraj, 2021) could be considered in future studies as some samples with low-quality DNA yield may contain plastid reads sufficient for DNA sequencing and subsequent analyses.

Our findings show that plastid DNA from herbarium specimens can be used as a ready source of samples for phylogenetic analysis (Särkinen *et al.*, 2012; Staats *et al.*, 2013; Bakker *et al.*, 2016). However, it was necessary to fine-tune the laboratory protocols to minimize damage of already degraded herbarium DNA. We also recommend a modification of the *in silico* pipeline to minimize data loss. Pooling multiple DNA extractions from each sample to increase the starting DNA yield before sequencing, or an additional target capture step, may also be necessary to achieve higher data quality (Andermann *et al.*, 2020), resulting in greater assembly coverage.

### The whole-plastome phylogeny resolves most main clades of *Diospyros*

The phylogenetic placement of several clades of *Diospyros* have historically been uncertain, due to the relatively few plastid loci used in previous studies; however, the use of whole-plastid genomes of over 100 *Diospyros* species for phylogenetic reconstruction has resulted in a robust and well-resolved phylogeny. The phylogenetic tree inferred from these data through maximum likelihood, unsurprisingly, has greater resolution than trees generated using subsets of one to seven genes (average bootstrap 95 versus 88 % in the six-locus dataset versus up to 90 % in other combination schemes; Table 3) with fewer short internodes and polytomies. The previous, poorly resolved *Diospyros* Clades VIII, IX and X (Duangjai *et al.*, 2009; Turner *et al.*, 2013*a*) are now well resolved in the whole-plastome tree.

Despite the use of entire plastome sequences, relationships along the backbone of Clade XI as well as the New Caledonian species within Clade III remain largely unresolved. The latter case has been discussed in the previous New Caledonian-focused study by Turner *et al.* (2016), who found that these species have undergone multiple bursts of diversification, and whole-plastome data did not provide sufficient phylogenetic information. Paun *et al.* (2016) suggest that nuclear single-nucleotide polymorphism (SNP) data are better suited for phylogenetic studies of this group. Several other species complexes remain unresolved, such as the *D. japonica–D. lotus* (Clade IX) and *D. ferrea* complexes (Clade III), and may benefit from a RADseq approach. *D. japonica* was treated as a synonym of *D. lotus* in several taxonomic accounts (Hiern, 1873; Forbes and Hemsley, 1889; Lecomte, 1930), and the two might be conspecific, in which case we do not expect further resolution. In contrast, *D. ferrea* has long been recognized as a taxonomically challenging and widespread species complex, having a range extending from West Africa to as far east as Hawaii (Pannell and White, 1988; Singh, 2005; Schatz *et al.*, 2013; Mestre Serra *et al.*, 2024, 2025). Seeds of species in this group are readily dispersed over long distances due to zoochory (Pannell and White, 1988; White, 1992), and the subsets of the complex have recently been divided into six species in mainland Africa and Madagascar (Mestre Serra *et al.*, 2024, 2025).

Due to the slow rate of evolution of the chloroplast genome (Clegg *et al.*, 1994), whole-plastome data have failed to resolve phylogenetic relationships in other plant groups, such as *Isodon* (Chen *et al.*, 2022), some clades of Ochnaceae (Schneider *et al.*, 2021), and *Quercus* (Pham *et al.*, 2017), all of which seem to have undergone rapid radiation, or extensive hybridization events in the case of *Quercus*. The New Caledonian *Diospyros* revealed discordance between the plastid phylogeny, distribution and morphology, suggesting that plastid capture due to hybridization might have contributed to the rapid evolution of this group (Turner *et al.*, 2016). Indeed, introgressive hybridization has been detected in multiple studies of *Diospyros* based on nuclear DNA data (Yonemori *et al.*, 2008; Turner *et al.*, 2013*b*; Paun *et al.*, 2016; Linan *et al.*, 2021). These findings suggest that even though the plastome is the most accessible resource for *Diospyros* phylogenetic studies, its slower rate of evolution and uniparental inheritance does not allow reconstruction of relationships in rapidly evolving and hybrid lineages. In these cases, fast-evolving nuclear markers should be employed to resolve relationships within problematic groups.

### The most variable gene regions provide an alternative path for phylogenetic studies of *Diospyros*

While complete plastome sequencing has been shown to provide robust information for *Diospyros* phylogenetic reconstruction in this and previous studies (Burke *et al.*, 2014; Eserman *et al.*, 2014; Givnish *et al.*, 2015; Burke *et al.*, 2016; Guo *et al.*, 2017; Twyford and Ness, 2017; Bi *et al.*, 2018; Gitzendanner *et al.*, 2018; Ye *et al.*, 2018; Lloyd Evans *et al.*, 2019; Stettler *et al.*, 2021; Mo *et al.*, 2022; Yang *et al.*, 2022), our results also highlight that similar topologies, although with somewhat lower support values, can be obtained by sequencing just a fraction of the plastome, targeting a handful of the most variable plastid regions in the genus, which could prove useful in cases where whole-plastome sequencing cannot be achieved. We found that the nucleotide diversity hotspots in the Ebenaceae plastid genomes are located in the LSC and the SSC. This finding is largely congruent with a comparative study of 45 *Diospyros* plastomes (Huang *et al.*, 2023) which focused on temperate Asian and New Caledonian species. However, our whole-plastome alignments show some minor differences, such as higher nucleotide diversity within the *rbcL* gene, which could be due to several factors, including (1) our larger set of taxonomically diverse taxa (114 taxa in our study vs 45 taxa in their study), (2) use of a different set of window sizes for the sliding window analysis (200, 600 and 1000 bp vs 600 bp), and (3) use of a smaller step size (50 vs 200 bp).

Multiple intergenic spacers, such as *ndhF–rpl32*, *rpl32–trnL* and *ccsA–ndhD*, have been identified both in this study and others (Dong *et al.*, 2012; Shaw *et al.*, 2014; Huang *et al.*, 2023) as hotspots of nucleotide diversity; however, the use of intergenic spacers alone in phylogenetic reconstruction can be error-prone during the alignment step, where there is much divergence. Mutations within non-coding intergenic spacers are not constrained by natural selection and as a result there can be highly variable sequences and long indels that are more likely to be erroneously aligned (Kelchner, 2000).

Among the gene regions identified in our study as good candidates for future phylogenetic work, *ycf1*, *matK* and *ndhF* have previously been identified as some of the most phylogenetically informative in angiosperms (Neubig *et al.*, 2009; Dong *et al.*, 2015; D’Agostino *et al.*, 2018; Amar, 2020; Chen *et al.*, 2020; Liu *et al.*, 2020; Li *et al.*, 2021; Huang *et al.*, 2023). For *Diospyros* in particular, the genes *atpB*, *matK*, *ndhF* and *rbcL* have been utilized in previous phylogenetic studies, along with *trnK* intron, *trnL* intron and *trnL–trnF* spacer (Duangjai *et al.*, 2006; Duangjai *et al.*, 2009; Turner *et al.*, 2013*a*; Linan *et al.*, 2019), but of them, only *matK* and *ndhF* are identified here as two of the ten most variable genes in our dataset. The other two genes, *rbcL* and *atpB*, are ranked as the 24th and 26th among 113 genes by the number of variable sties. Despite the comparatively low level of variability in these genes, they have a long history of use in plant phylogenetic studies and thus are widely available for comparison (Chase *et al.*, 1993; Hoot *et al.*, 1995; Savolainen *et al.*, 2000; Shaw *et al.*, 2005; Prince, 2015). Despite being the most mutation-rich plastid protein-coding gene in our dataset, *ycf1* has not been widely used due to its length (5562 bp on average), which far exceeds the 1000-bp DNA sequences that Sanger sequencing can produce without intermediate sequencing primers. Use of *ycf1* in Orchidaceae (Neubig *et al.*, 2009), *Prunus* (Amar, 2020) and *Astragalus* (Dastpak *et al.*, 2018) has shown the potential for this gene. Dong *et al.* (2015) designed two pairs of primers amplifying two subregions within the *ycf1* gene that were successfully used as DNA barcodes in 357 plant species. Other relatively cheap long-read solutions combined with long-range PCR could be applied to the sequencing of *ycf1* in *Diospyros* for phylogenetic studies.

When combining the most variable genes with the existing six loci (Dataset IV), our analysis revealed that the inclusion of these genes significantly improved both the bootstrap support values and the topological similarity with the reference plastome tree. Among these scenarios, the addition of the *ycf1* gene to the core six plastid markers provided the most substantial improvement. In cases when the entire plastome cannot be obtained, the *ycf1* gene should be included with the core six traditional plastid markers as a viable alternative for constructing the phylogenetic framework of *Diospyros*. Furthermore, although the markers *rbcL* and *atpB* are more conserved, they remain valuable for elucidating the evolutionary history at broader phylogenetic scales (Savolainen *et al.*, 2000).

### An updated phylogenetic tree of *Diospyros* using the most commonly available loci (wider six-locus tree)

Although the complete plastome could not be generated for most of our samples, we were able to assemble the six commonly used plastid loci in Ebenaceae (*atpB*, *ndhF*, *rbcL*, *trnK* (including *matK*), *trnL–trnF* and *trnS–trnG*) from 214 samples (out of 324), which allowed us to combine our data with existing Sanger sequencing data to reconstruct an updated multi-locus phylogeny of the family focusing on *Diospyros*. Even though the concatenated alignment of these six loci has a length of just under 10 000 bp, it comprises the largest set of *Diospyros* and Ebenaceae samples and taxa to date (Dataset V, 527 samples from over 263 taxa). This phylogeny provides the most comprehensive evidence for many clades and taxon placements, leading us to better understand the evolution of *Diospyros*.

In general, relationships between the ten clades of *Diospyros* produced using the core six-locus tree (Dataset V) are similar to those previously published (Duangjai *et al.*, 2009; Turner *et al.*, 2013b) and our newly constructed plastome phylogeny (Dataset II). Of particular note is the contrast between Clade VIII in Dataset V, which is sister to Clade IX (BS 98 %), and Dataset II, where Clade VIII is sister to Clade X (BS 100 %). The phylogeny from Dataset V has lower bootstrap support within some clades, such as Clades III and XI. This might indicate rapid bursts of evolution, such that only a small number of mutations have accumulated through time, and these rare mutations might not always be sampled in pseudoreplications during bootstrap tree reconstruction (Felsenstein, 1985). The poorly supported Clade XI backbone present in the wider six-locus tree (BS as low as 18 %) was better resolved when using the whole-plastid data and support values are found to be higher (BS 75–80 %).

Unlike some other plant groups where major clades are usually geographically defined, such as *Lupinus* (Fabaceae) (Ainouche and Bayer, 1999), Musaceae (Janssens *et al.*, 2016) and *Quercus* (Fagaceae) (Pham *et al.*, 2017), *Diospyros* Clades III, IX and XI contain species with wide or disjunct distribution. Southeast Asian species are found in all major clades of *Diospyros* with the exception of Clades VIII (only African species) and IV (only African and South American species). Duangjai *et al.* (2009), who tested the dispersal-vicariance (DIVA) of *Diospyros*, found that the explanation of the current biogeography of the genus can only be dispersal. They also found that Clade XI, which is the most geographically dispersed clade, with species found in Australasia, Indo-Malay and Neotropics, originated in Eurasia and underwent long-distance migration to other continents.

At the species level, this phylogeny not only resolves the relationships among most *Diospyros* lineages but also identifies closely related species complexes that need further resolution and may benefit from sequencing approaches targeting nuclear genes, e.g. using RADseq or the Angiosperm353 probe set (Johnson *et al.*, 2019). These complex groups include *D. maritima–D. undulata*, *D. eriantha–D. rufogemmata*, *D. sylvatica–D. venosa*, *D. montana–D. rhombifolia*, *D. japonica–D. lotus*, *D. collinsiae–D. retrofracta* and *D. ferrea* complexes (ordered from top down in Supplementary Data Tree S1). These should be the focus of further studies using more comprehensive datasets of genomic, taxonomic and morphological data.

### Conclusions

We have more than doubled the complete plastome sequences reported for Ebenaceae, including 50 % newly reported species and detailed sampling within species complexes to help resolve their identity. This study generated whole-plastid genomes for 74 species (110 samples) of *Diospyros*, two species of *Euclea* and three species of *Royena*. This has yielded the best-supported phylogenetic backbone for the family to date and represents a step change in our knowledge of Ebenaceae evolutionary patterns.

Combining data extracted from our plastome analysis with data on the six plastid genes commonly sequenced for the family, we have further added phylogenetic knowledge resolving some of the uncertain relationships in the family. There remains uncertainty in two clades that may have undergone rapid radiation, resulting in few reliable DNA markers to resolve their relationships. Where resources to sequence whole plastomes are not available we recommend the addition of *ycf1* to greatly increase resolution when combined with the six commonly used plastid markers in *Diospyros*.

The plastome data and phylogenetic trees generated from this study represent a leap forward in our understanding of relationships within the genus and will allow us to address biogeographic and taxonomic questions regarding *Diospyros* species across Southeast Asia and facilitate molecular identification in this genus. Additionally, this study offers a foundational phylogenetic framework for future taxonomic studies of Southeast Asian *Diospyros* in a global context. There remains a lack of data on Indian subcontinent taxa in the family. Future research aims to expand the phylogenetic framework by incorporating more species and focusing on resolving complex species identified in this study, either through whole-plastome data or using the core six loci along with the *ycf1* gene. For groups or clades that are rapidly evolving or may have undergone interspecies introgression or hybridization, it will be important to include multiple nuclear markers in the analysis.

## Supplementary Material

mcag084_Supplementary_Data

## SUPPLEMENTARY DATA

Supplementary data are available at *Annals of Botany* online and consist of the following. Table S1: DNA sequences generated in this study and the availability of plastome assemblies. Table S2: DNA accessions used in this study with collector and locality details. Table S3: details of nucleotide alignment of Dataset 1 (Tab 1: number and proportion of variable sites; Tabs 2–4: nucleotide diversity based on 200, 600 and 1000 bp sliding windows, respectively; Tab 5: position of gene annotations on the alignment). Table S4: average bootstrap support, total tree length and weighted Robinson-Foulds (wRF) distance of phylogenies in this analysis. Tree S1: estimated maximum likelihood phylogenetic tree based on the commonly used six loci (*atpB*, *ndhF*, *rbcL*, *trnK–matK*, *trnL–trnF* and *trnS–trnG*) (Dataset V). all_trees.trees: the original tree files in the Newick format with their file names corresponding to the dataset names in Table 1.
