# Supplementary material for: A global phylogenetic framework for Diospyros L. (Ebenaceae, Ericales): enhanced taxon and sample coverage through NGS, Sanger sequencing and herbariomics
Source: Ann Bot. 2026 Apr 8;138(2):442–61. doi: 10.1093/aob/mcag084 (PMC13454686; doi:10.1093/aob/mcag084)
Supplement: Supplementary file 1 [file mcag084_supplementary_data.zip › Tree S1.pdf]

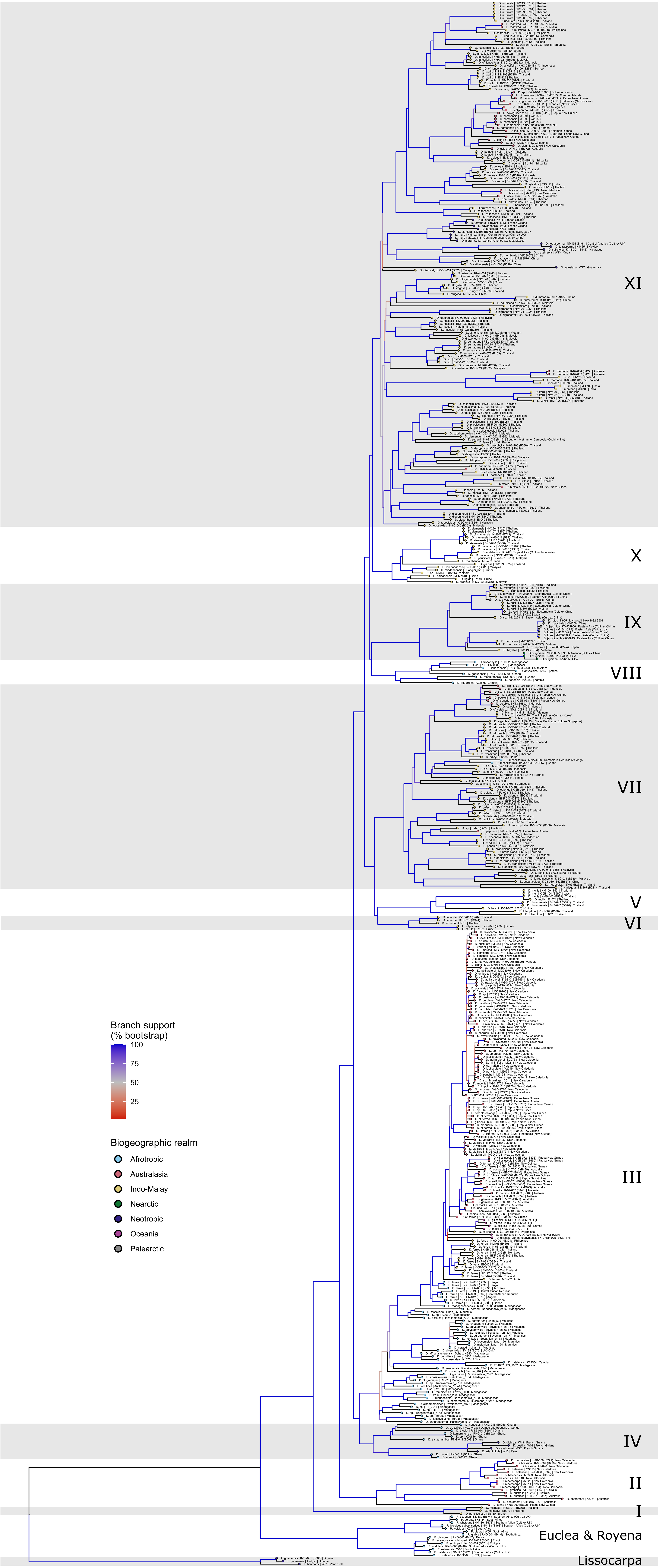

XI
X
IX
VIII
VII
V
VI
III
IV
II
I
Euclea & Royena
Lissocarpa
Branch support
(% bootstrap)
100
75
50
25
Biogeographic realm
Afrotropic
Australasia
Indo-Malay
Nearctic
Neotropic
Oceania
Palearctic
